# Gestrinone, lipedema, and the limits of theoretical extrapolation in clinical practice: a critical narrative review

**DOI:** 10.1590/1677-5449.202502402

**Published:** 2026-07-20

**Authors:** Marcelo Fernandes Lima

**Affiliations:** 1 Clínica Dr. Marcelo Lima – Medicina Vascular, Manaus, AM, Brasil.

**Keywords:** lipedema, adipose tissue, hormone therapy, gestrinone, evidence-based medicine

## Abstract

Lipedema is a chronic inflammatory disorder of adipose tissue that predominantly affects women and is characterized by disproportionate subcutaneous fat accumulation, pain, spontaneous bruising, and significantly impaired quality of life. Although hormonal influences on its pathophysiology have been increasingly recognized, evidence-based treatment options remain limited. Recently, gestrinone has been proposed as an off-label therapy based on hormonal mechanistic hypotheses. A critical narrative review of the literature was conducted using international databases to identify clinical or pharmacological evidence supporting its use in lipedema. No clinical trials, observational studies, case series, or case reports directly evaluating this therapy were identified. Existing proposals rely exclusively on theoretical extrapolations lacking clinical validation. Therefore, current evidence does not support the use of gestrinone in the treatment of lipedema, and its use should be considered experimental until adequately designed clinical studies are available.

## INTRODUCTION

Lipedema is a chronic inflammatory disease of adipose tissue, predominantly affecting women, characterized by a symmetrical and disproportionate accumulation of subcutaneous fat. Clinical manifestations often include pain, tissue tenderness, spontaneous bruising, functional limitations, and mood changes with a tendency toward depressive states, significantly impacting quality of life. Despite increasing recognition, lipedema remains underdiagnosed, and evidence-based options for its treatment are limited.^1-8^

Recent evidence points to complex interactions across adipose tissue dysfunction, genetic polymorphisms, chronic inflammation, microvascular alterations, and hormonal regulation as responsible for the anomalous fat distribution which characterizes lipedema.^5-8^ Onset or progression of lipedema has consistently been observed to occur during periods of hormonal fluctuation, such as puberty, pregnancy, and menopause, suggesting a relevant role for estrogen signaling and intracrine hormone metabolism in the pathophysiology of this disease.^6-8^

Within this context, off-label pharmacotherapeutic interventions seeking to modulate hormone levels have elicited great interest in clinical practice. Chief among these is gestrinone, a synthetic steroid with antiprogestogen, antiestrogen, and androgenic effects which is widely used in the treatment of estrogen-dependent gynecological conditions, such as endometriosis.^9-11^

The aim of this critical narrative review was to examine the available literature on the use of gestrinone in lipedema, assess the nature and quality of the existing evidence, and distinguish theoretical proposals from validated clinical data.

## MATERIAL AND METHODS

A critical narrative review of the literature was conducted to identify clinical, observational, or mechanistic evidence supporting the use of gestrinone in the treatment of lipedema. It bears stressing that this is not a systematic review, and that the conclusions presented herein are limited to describing the absence of direct clinical evidence of a therapeutic effect of gestrinone in lipedema and highlighting the risks of mechanistic extrapolation across distinct conditions without evidence to corroborate such an approach.

### Search strategy

PubMed (via MEDLINE), Embase, Scopus, Web of Science Core Collection, Cochrane Library, and Google Scholar databases were searched. The time interval considered was from January 1980 to January 30, 2026 (the date of the last search performed). No restrictions on language or study design were applied during the initial search phase, considering the expected scarcity of direct clinical evidence.

The following search query was used for the PubMed database: (lipedema OR lipoedema OR "painful fat syndrome") AND (gestrinone OR gestrinona). Given the likely absence of direct clinical studies, additional expanded searches were conducted using the following combined terms: (lipedema OR lipoedema) AND (hormone therapy OR antiestrogen OR antiprogestin OR progestin OR androgenic steroid OR hormonal modulation) e (gestrinone) AND (adipose tissue OR inflammation OR estrogen receptor OR progesterone receptor). Equivalent strategies were adapted as appropriate to the other databases.

### Management of records and duplicates

All records retrieved were subjected to manual verification for duplicates. Duplicates across databases were identified by comparing title, authors, year of publication, and DOI. Duplicate records were removed before screening by title and abstract.

### Screening process

Study selection was a three-step process: identification – total number of records retrieved from the databases; screening by title and abstract – records irrelevant to the topic of interest were excluded; and full-text reading – eligibility was assessed in detail.

The results of the database searches were compiled, and the principal investigator screened titles and abstracts, and then screened full-text articles for selection on the basis of the inclusion and exclusion criteria. If a consensus was not reached for final articles, a second independent reviewer would be consulted.

### Eligibility criteria

Studies were considered eligible if they met at least one of the following criteria: direct assessment of the use of gestrinone in the treatment of patients with lipedema; investigation of pharmacological, metabolic, hormonal, or anti-inflammatory effects of gestrinone which may be relevant to adipose tissue metabolism; mechanistic discussions or hypotheses relating hormonal modulation to lipedema.

The following studies were excluded: reports with a purely cosmetic focus; opinion articles not based on scientific evidence; publications unrelated to lipedema or relevant hormonal mechanisms; and publications for which no full text was available.

### Data synthesis

Due to the absence of direct clinical evidence, the data were analyzed using a qualitative narrative approach, with an emphasis on the distinction between empirical clinical evidence, indirect mechanistic evidence, and theoretical proposals. Given the narrative scope of the review, no meta-analysis or formal risk of bias assessment was performed.

## RESULTS

A total of 115 records were identified in the databases. After removal of duplicate references, 89 articles were screened by title and abstract, of which 61 were excluded due to irrelevance to the topic of interest. Twenty-eight articles were read in full. Thirteen of these were excluded because they did not address lipedema, did not report a relationship with gestrinone or relevant hormonal mechanisms, or because they had an exclusively cosmetic focus.

No clinical trials, observational studies, case series, or case reports were identified that directly evaluated the use of gestrinone in patients with lipedema.^12,13^

Only one peer-reviewed publication explicitly proposed the use of gestrinone for the treatment of lipedema. This was a mechanistic narrative review that discussed possible hormonal links between lipedema and gynecological disorders and suggested potential benefits of gestrinone and drospirenone.^14^

Several studies were identified that evaluated gestrinone in other estrogen-dependent conditions, especially endometriosis, describing its endocrine, metabolic, and anti-inflammatory effects.^8-10^ Although these studies provide relevant pharmacological information, none addressed lipedema or primary adipose tissue disorders. In parallel, several reviews and mechanistic studies on lipedema support a role of inflammation and hormonal signaling in its pathogenesis, without, however, evaluating specific hormonal therapies such as gestrinone.^1-8^

## DISCUSSION

This critical narrative review demonstrates the absence of direct clinical evidence to support the use of gestrinone in the treatment of lipedema. Despite growing clinical interest, no empirical or evidence-based data have been identified to justify its therapeutic use in this condition.^11-13^

Lipedema is increasingly understood as a disorder of adipose tissue influenced by hormones, involving an imbalance of estrogen receptors, intracrine estrogen metabolism, genetic polymorphisms, and chronic inflammation. These findings offer biological plausibility for further investigation of hormonal therapies; however, plausibility does not equate to clinical efficacy or even safety.^1-8^

Gestrinone has potent antiprogestogen and antiestrogen effects, as well as androgenic properties, with documented impact on lipid metabolism and endocrine function in patients with endometriosis. Extrapolating these effects to the treatment of lipedema, while theoretically appealing, remains speculative. In addition to the lack of direct clinical evidence of efficacy, any methodological evaluation of gestrinone use in lipedema must necessarily consider its safety profile. When used patients with endometriosis it has been reported to have adverse androgenic effects, including acne, hirsutism, seborrhea, changes in vocal timbre, and alopecia, as well as relevant metabolic abnormalities, including reduced HDL-cholesterol and increased LDL-cholesterol levels. These impacts on the lipid profile and hypothalamic-pituitary-ovarian axis are well documented in the gynecological population, but no data exist to date in patients with lipedema. Extrapolating these findings to the population with lipedema requires empirical validation, especially considering that this condition has a documented microvascular component and is frequently associated with metabolic issues. There are no studies specifically evaluating the safety, vascular impact, or metabolic effects of gestrinone in patients with lipedema. Therefore, any proposed therapeutic use in this context is based exclusively on indirect inference from other clinical indications. From a methodological standpoint, the use of a known androgen which has potential impacts on lipid profile in a population with vascular dysfunction, with no prior demonstration of clinical benefit, represents a pharmacological extrapolation unsupported by specific evidence. In the absence of adequately designed clinical trials, the risk-benefit ratio remains undefined, and this use should be considered experimental.^8-10,13^

The only publication that proposed the use of gestrinone in the treatment of lipedema is a purely hypothesis-generating contribution to the literature, not clinical evidence. This was a mechanistic, non-systematic narrative review that proposed a hypothetical pathophysiological model for lipedema centered on dysfunction of the estrogen-adipose tissue axis, particularly an imbalance between α and β estrogen receptors. To support and suggest the use of gestrinone in lipedema, the authors repeatedly state that lipedema and gynecological diseases share a common hormonal pathway characterized by estrogen dominance, progesterone resistance, and local inflammation. The central issue to be analyzed is that no study cited in the review analyzes lipedematous adipose tissue and pathological endometrial tissue in the same design, demonstrates that these pathways are actively present in lipedema, or directly compares local hormonal profiles between the two diseases.^14^

It should also be noted that, particularly in the case of the alleged progesterone resistance shared between lipedema and gynecological diseases, the authors reach this conclusion based on an article published in 1998, writing that “in lipedema, progesterone resistance has also been documented” then citing the aforementioned article.^15^ For this statement to be scientifically valid, the cited article would need to either study patients with lipedema or analyze adipose tissue clearly identified as lipedematous. However, O’Brien et al.^15^ evaluated progesterone resistance in reproductive tissues (mainly the endometrium) associated with conditions such as endometriosis and in a gynecological context; they did not document progesterone resistance in lipedema. This is questionable and scientifically inadequate, because the authors assume that lipedema shares hormonal mechanisms with endometriosis, making an undeclared extrapolation and presenting a hypothesis by analogy as documented fact. This creates a false evidence base, eliciting a perception that there is already molecular evidence of progesterone resistance in lipedema – which is untrue – and lending false legitimacy to proposed hormonal treatments based on narratives of a common mechanism that is, at the very least, unproven.

Even in narrative reviews, which are known to be less rigorous than systematic reviews, fidelity to the original content of the sources must be observed, and a clear distinction must be made between direct evidence and theoretical inference. The use of terms such as “has been documented,” “is consistently observed,” “represents a common pathway,” and “suggests a therapeutic rationale” is incompatible with the actual level of evidence, which is still hypothetical, indirect, and analogical.^14^

Narrative reviews do not establish causality, do not validate therapeutic efficacy, and do not allow for clinical recommendations, especially when they extrapolate from indirect data. Despite the authors acknowledging this limitation, they still propose the use of gestrinone and drospirenone in lipedema, which represents a methodological incongruity.^14^

Treating such proposals as therapeutic validation carries the risk of conflating theoretical reasoning with evidence-based practice. This distinction is essential to preserve scientific rigor and patient safety.^13^

## CONCLUSION

Current evidence does not support the use of gestrinone in the treatment of lipedema. The available literature is limited to indirect pharmacological data and theoretical discussions, lacking any clinical validation. Although hormonal mechanisms play a modulating role in the pathophysiology of lipedema, translating this knowledge into therapeutic interventions requires rigorous clinical investigation. The clear distinction between hypothesis and evidence remains key to guiding both clinical practice and future research.

The findings of the present review conclude that there is no scientific evidence to support the use of gestrinone in lipedema, highlighting an urgent need for well-designed clinical studies investigating hormonal modulation in this condition. Until such studies are available, the use of gestrinone in lipedema should be considered experimental and restricted to research protocols which have received ethical approval.
