# Supplementary material for: Gestrinone, lipedema, and the limits of theoretical extrapolation in clinical practice: a critical narrative review
Source: J Vasc Bras. 2026 Jul 20;25:e20250240. doi: 10.1590/1677-5449.202502402 (PMC13441213; doi:10.1590/1677-5449.202502402)
Supplement: Translation

# Translation: Gestrinona, lipedema e os limites da extrapolação teórica na prática clínica: uma revisão narrativa crítica

**DOI:** 10.1590/1677-5449.202502401

**Authors:** Marcelo Fernandes Lima

**Affiliations:** 1 Clínica Dr. Marcelo Lima – Medicina Vascular, Manaus, AM, Brasil.

**Keywords:** lipedema, tecido adiposo, terapia hormonal, gestrinona, medicina baseada em evidências

## Abstract

O lipedema é uma doença inflamatória crônica do tecido adiposo caracterizada por acúmulo desproporcional de gordura subcutânea, dor e equimoses espontâneas, que acomete predominantemente mulheres e impacta a qualidade de vida. Apesar do crescente reconhecimento da influência hormonal nessa doença, as opções terapêuticas baseadas em evidências permanecem limitadas. Recentemente, a gestrinona tem sido proposta como tratamento *off-label*, sendo fundamentada em hipóteses mecanicistas hormonais. Foi realizada uma revisão narrativa crítica da literatura, utilizando bases de dados internacionais, com o objetivo de identificar evidências clínicas ou farmacológicas que sustentassem seu uso no tratamento do lipedema. Não foram encontrados ensaios clínicos, estudos observacionais, séries de casos ou relatos de caso avaliando diretamente essa terapêutica. As propostas existentes baseiam-se exclusivamente em extrapolações teóricas, sem validação clínica. Assim, as evidências atuais não sustentam o uso da gestrinona no tratamento do lipedema, devendo seu uso ser considerado experimental até a realização de estudos clínicos adequadamente delineados.

## INTRODUÇÃO

O lipedema é uma doença inflamatória crônica do tecido adiposo caracterizada por acúmulo simétrico e desproporcional de gordura subcutânea, sendo predominante em mulheres. Clinicamente, caracteriza-se por dor, sensibilidade tecidual, equimoses espontâneas, limitação funcional e alterações do humor com tendências a estados depressivos, impactando significativamente a qualidade de vida. Apesar do reconhecimento crescente, o lipedema permanece subdiagnosticado, e suas opções terapêuticas, baseadas em evidência científica, são limitadas^1-8^ .

Evidências recentes apontaram para interações complexas entre disfunção do tecido adiposo, polimorfismos genéticos, inflamação crônica, alterações microvasculares e regulação hormonal como responsáveis pela distribuição anômala de gordura^5-8^ . Observações clínicas consistentes identificaram início ou progressão da doença em períodos de flutuação hormonal — como puberdade, gestação e menopausa —, sugerindo papel relevante da sinalização estrogênica e do metabolismo hormonal intrácrino na fisiopatologia do lipedema^6-8^ .

Nesse contexto, terapêuticas farmacológicas *off-label* voltadas à modulação hormonal têm despertado interesse na prática clínica. Entre elas, destaca-se a gestrinona, um esteroide sintético com propriedades antiprogestagênicas, antiestrogênicas e androgênicas, utilizada com frequência no tratamento de condições ginecológicas estrogênio-dependentes, como a endometriose^9-11^ .

O objetivo desta revisão narrativa crítica foi examinar a literatura disponível sobre o uso da gestrinona no lipedema, avaliar a natureza e a qualidade das evidências existentes e diferenciar propostas teóricas de dados clínicos validados.

## MÉTODOS

Foi realizada uma revisão narrativa crítica da literatura com o objetivo de identificar evidências clínicas, observacionais ou mecanicistas que sustentassem o uso da gestrinona no tratamento do lipedema. É necessário enfatizar que não se trata de revisão sistemática e que as conclusões apresentadas são limitadas à ausência de evidência clínica direta de ação terapêutica da gestrinona no lipedema e aos riscos da extrapolação mecanicista entre doenças distintas sem evidências que corroborem essa conduta.

### Estratégia de busca

A busca foi conduzida nas bases de dados PubMed/MEDLINE, Embase, Scopus, Web of Science Core Collection, Cochrane Library e Google Scholar. O intervalo temporal considerado foi de janeiro de 1980 até 30 de janeiro de 2026, data da última busca realizada. Não foram aplicadas restrições quanto ao idioma ou ao desenho do estudo na etapa inicial da busca, considerando-se a expectativa de escassez de evidência clínica direta.

Foi utilizada a seguinte estratégia na base PubMed: (lipedema OR lipoedema OR "painful fat syndrome") AND (gestrinone OR gestrinona). Considerando a provável ausência de estudos clínicos diretos, foram realizadas buscas adicionais ampliadas com os seguintes termos combinados: (lipedema OR lipoedema) AND (hormone therapy OR antiestrogen OR antiprogestin OR progestin OR androgenic steroid OR hormonal modulation) e (gestrinone) AND (adipose tissue OR inflammation OR estrogen receptor OR progesterone receptor). Estratégias equivalentes foram adaptadas às demais bases.

### Gerenciamento de registros e duplicatas

Todos os registros identificados foram submetidos à verificação manual de duplicatas. Duplicações entre bases foram identificadas por comparação de título, autores, ano de publicação e DOI. Os registros duplicados foram removidos antes da triagem por título e resumo.

### Processo de triagem

A seleção dos estudos ocorreu em três etapas: identificação – total de registros recuperados nas bases; triagem por título e resumo – exclusão por irrelevância temática; e leitura de texto completo – avaliação detalhada de elegibilidade.

Os resultados das buscas nas bases de dados foram compilados e o autor principal analisou os títulos e resumos e, em seguida, procedeu à leitura na íntegra dos artigos elegíveis para seleção com base nos critérios de inclusão e exclusão. Caso não chegasse a um consenso para os artigos finais, um segundo revisor independente seria consultado.

### Critérios de elegibilidade

Foram considerados elegíveis estudos que atenderam a pelo menos um dos seguintes critérios: avaliação direta do uso da gestrinona no tratamento de pacientes com lipedema; investigação de efeitos farmacológicos, metabólicos, hormonais ou anti-inflamatórios da gestrinona com relevância potencial para o metabolismo do tecido adiposo; discussões mecanicistas ou hipóteses relacionando modulação hormonal e lipedema.

Foram excluídos relatos puramente estéticos, artigos de opinião sem fundamentação científica, publicações sem relação com lipedema ou com mecanismos hormonais relevantes e textos aos quais não se tinha acesso ao conteúdo integral.

### Síntese dos dados

Devido à ausência de evidência clínica direta, os dados foram analisados por abordagem narrativa qualitativa, com ênfase na distinção entre evidência clínica empírica, evidência mecanicista indireta e propostas teóricas. Não foi realizada metanálise ou avaliação formal de risco de viés, dado o escopo narrativo da revisão.

## RESULTADOS

Foram identificados 115 registros nas bases de dados. Após a remoção de referências duplicadas, 89 artigos foram submetidos à triagem por título e resumo, dos quais 61 foram excluídos por irrelevância temática e 28 foram avaliados em texto completo. Desses, 13 foram excluídos por não abordarem lipedema, não apresentarem relação com gestrinona e/ou mecanismos hormonais relevantes, ou por terem foco exclusivamente estético.

Não foram identificados ensaios clínicos, estudos observacionais, séries de casos ou relatos de caso que avaliassem diretamente o uso da gestrinona em pacientes com lipedema^12,13^ .

Apenas uma publicação revisada por pares propôs explicitamente o uso da gestrinona para o tratamento do lipedema. Trata-se de uma revisão narrativa mecanicista que discute possíveis vínculos hormonais entre lipedema e distúrbios ginecológicos, sugerindo potenciais benefícios da gestrinona e da drospirenona^14^ .

Foram identificados diversos estudos que avaliaram a gestrinona em outras condições estrogênio-dependentes, especialmente a endometriose, descrevendo seus efeitos endócrinos, metabólicos e anti-inflamatórios^8-10^ . Embora esses estudos forneçam informações farmacológicas relevantes, nenhum deles aborda lipedema ou distúrbios primários do tecido adiposo. Paralelamente, revisões e estudos mecanicistas sobre lipedema sustentam o papel da inflamação e da sinalização hormonal, sem avaliar terapias hormonais específicas, como a gestrinona^1-8^ .

## DISCUSSÃO

Esta revisão narrativa crítica demonstra a ausência de evidência clínica direta que sustente o uso da gestrinona no tratamento do lipedema. Apesar do crescente interesse clínico, não foi identificado nenhum dado empírico ou estudo com embasamento em evidências que justifique sua aplicação terapêutica nessa condição^11-13^ .

O lipedema vem sendo cada vez mais compreendido como um distúrbio do tecido adiposo influenciado por hormônios, com envolvimento de desequilíbrio de receptores estrogênicos, metabolismo intrácrino do estrogênio, polimorfismos genéticos e inflamação crônica. Esses achados oferecem plausibilidade biológica para a investigação de terapias hormonais, porém a plausibilidade não equivale à eficácia e/ou à segurança clínica^1-8^ .

A gestrinona apresenta efeitos antiprogestagênicos e antiestrogênicos potentes, além de propriedades androgênicas, com impacto documentado sobre o metabolismo lipídico e a função endócrina em pacientes com endometriose. A extrapolação desses efeitos para o tratamento do lipedema, embora teoricamente atraente, permanece especulativa. Além da inexistência de evidência de eficácia clínica direta, a avaliação metodológica do uso da gestrinona no lipedema deve necessariamente considerar seu perfil de segurança. Seu uso em pacientes com endometriose teve efeitos androgênicos adversos, como acne, hirsutismo, seborreia, alterações de timbre vocal e alopecia, bem como alterações metabólicas relevantes, incluindo redução de lipoproteína de alta densidade e aumento de lipoproteína de baixa densidade. Tais alterações do perfil lipídico e do eixo hipotálamo-hipófise-ovariano são documentadas nessa população ginecológica, não existindo dados em pacientes com lipedema até a presente data. A extrapolação desses dados para a população com lipedema carece de validação empírica, sobretudo considerando que essa condição apresenta componente microvascular documentado e associação frequente com alterações metabólicas. Não há estudos avaliando especificamente segurança, impacto vascular ou efeitos metabólicos da gestrinona em pacientes com lipedema. Portanto, qualquer proposição terapêutica nesse contexto baseia-se exclusivamente em inferência indireta a partir de outras indicações clínicas. Em termos metodológicos, a utilização de um fármaco com perfil androgênico e potencial impacto no perfil lipídico em uma população com disfunção vascular sem demonstração prévia de benefício clínico representa uma extrapolação farmacológica não sustentada por evidência específica. Na ausência de ensaios clínicos adequadamente delineados, a relação risco-benefício permanece indefinida, devendo seu uso ser considerado experimental^8-10,13^ .

A única publicação que propõe o uso da gestrinona no tratamento do lipedema constitui uma contribuição geradora de hipóteses e não uma evidência clínica. Trata-se de uma revisão narrativa mecanicista, não sistemática, que propõe um modelo fisiopatológico hipotético para o lipedema, centrado na disfunção do eixo estrogênio-tecido adiposo, particularmente no desequilíbrio entre receptores de estrogênio α e β. Para embasar e sugerir o uso da gestrinona no lipedema, os autores afirmam repetidamente que lipedema e doenças ginecológicas compartilham uma via comum hormonal, caracterizada por dominância estrogênica, resistência à progesterona e inflamação local. A questão central a ser analisada é que nenhum estudo citado no artigo analisa tecido adiposo de lipedema e tecido endometrial patológico no mesmo desenho, demonstra que essas vias estão ativamente presentes no lipedema ou compara diretamente perfis hormonais locais entre as doenças^14^ .

É necessário, ainda, atentar-se ao fato de que, particularmente no caso da alegação de resistência à progesterona compartilhada entre lipedema e doenças ginecológicas, os autores chegaram a essa conclusão com base em um artigo publicado em 1998, declarando que “no lipedema, a resistência à progesterona também foi documentada”, referenciando então o artigo citado^15^ . Para que essa afirmação fosse cientificamente válida, o artigo citado precisaria ter estudado pacientes com lipedema ou analisado tecido adiposo claramente identificado como lipedematoso. No entanto, O’Brien et al.^15^ avaliaram resistência à progesterona em tecidos reprodutivos, principalmente no endométrio, associada a condições como endometriose e em contexto ginecológico, não documentando resistência à progesterona no lipedema. Essa informação é questionável e cientificamente inadequada, porque os autores assumem que o lipedema compartilha mecanismos hormonais com a endometriose, fazendo uma extrapolação não declarada e transformando uma hipótese, por analogia, em um fato documentado. Tal conduta cria uma falsa base de evidência, induzindo à percepção de que já existe evidência molecular de resistência à progesterona no lipedema, o que não é verídico e possibilita uma falsa legitimação de propostas terapêuticas hormonais baseadas em narrativas de mecanismo comum no mínimo não comprovado.

Mesmo em revisões narrativas que são sabidamente menos rigorosas que revisões sistemáticas, deve-se observar fidelidade ao conteúdo original das fontes e fazer distinção clara entre evidência direta e inferência teórica. A utilização de termos como “foi documentada”, “é consistentemente observado”, “representa uma via comum” e “sugere uma racionalidade terapêutica” é incompatível com o nível real de evidência, que é hipotético, indireto e analógico^14^ .

Revisões narrativas não estabelecem causalidade, não validam eficácia terapêutica e não permitem recomendações clínicas, especialmente quando extrapolam dados indiretos. Apesar de os autores reconhecerem essa limitação, ainda assim, propõem o uso de gestrinona e drospirenona no lipedema, o que representa uma incongruência metodológica^14^ .

Tratar tais propostas como validação terapêutica implica risco de confundir raciocínio teórico com prática baseada em evidência. Essa distinção é essencial para preservar o rigor científico e a segurança do paciente^13^ .

## CONCLUSÃO

As evidências atuais não sustentam o uso da gestrinona no tratamento do lipedema. A literatura disponível limita-se a dados farmacológicos indiretos e discussões teóricas sem validação clínica. Embora os mecanismos hormonais tenham um papel modulador na fisiopatologia do lipedema, a tradução desse conhecimento em intervenções terapêuticas exige investigação clínica rigorosa. A distinção clara entre hipótese e evidência permanece fundamental para orientar tanto a prática clínica quanto a pesquisa futura.

Os achados desta revisão concluem não haver evidência científica que sustente o uso da gestrinona no lipedema, com necessidade urgente de estudos clínicos bem delineados que investiguem a modulação hormonal no lipedema. Até que tais pesquisas estejam disponíveis, o uso da gestrinona no lipedema deve ser considerado experimental e restrito a protocolos de pesquisa com aprovação ética.
